# Higher prevalence of idiopathic normal pressure hydrocephalus‐like MRI features in progressive supranuclear palsy: An imaging reminder of atypical parkinsonism

**DOI:** 10.1002/brb3.2884

**Published:** 2023-01-12

**Authors:** Mu‐Hui Fu, Chih‐Cheng Huang, Kay L. H. Wu, Ying‐Fa Chen, Yu‐Chih Kung, Cheng‐Chang Lee, Jia‐Shou Liu, Min‐Yu Lan, Yung‐Yee Chang

**Affiliations:** ^1^ Department of Neurology, Kaohsiung Chang Gung Memorial Hospital Chang Gung University College of Medicine Kaohsiung Taiwan; ^2^ Center for Parkinson's Disease Kaohsiung Chang Gung Memorial Hospital Kaohsiung Taiwan; ^3^ School of Medicine National Sun Yat‐Sen University Kaohsiung Taiwan; ^4^ Institute for Translational Research in Biomedicine Kaohsiung Chang Gung Memorial Hospital Kaohsiung Taiwan; ^5^ Department of Senior Citizen Services National Tainan Institute of Nursing Tainan Taiwan; ^6^ Department of Nursing Meiho University Pingtung County Taiwan; ^7^ Department of Radiology Kaohsiung Chang Gung Memorial Hospital and Chang Gung University College of Medicine Kaohsiung Taiwan

**Keywords:** atypical parkinsonism, idiopathic normal pressure hydrocephalus, Parkinson's disease, progressive supranuclear palsy

## Abstract

**Objectives:**

The classic triad of idiopathic normal pressure hydrocephalus (NPH) encompass gait disturbance, cognitive impairment, and urinary incontinence. These symptoms overlap with parkinsonism but with distinct treatment. Lacking applicable differentiation also hampers the prediction to therapeutic response. Here, we try to clarify this issue among different Parkinsonian syndromes and propose some innovative thinking while approaching a patient with parkinsonism and hydrocephalus concomitantly.

**Methods:**

Twenty‐four patients with clinical probable multiple system atrophy (MSA), 34 with probable progressive supranuclear palsy (PSP), and 58 with sex‐ and age‐matched Parkinson's disease (PD) were enrolled. Evans’ index (EI), callosal angle (CA), antero‐posterior (AP) diameter of the midbrain, length of the midbrain tegmentum diameter (MB^Tegm^), and disproportionately enlarged subarachnoid space hydrocephalus (DESH) were evaluated using the conventional MRI. Logistic regression was applied to identify the independent variables in hydrocephalus.

**Results:**

Patients with PSP had higher mean EI than those with MSA and PD. Around 38.2% of patients with PSP had accompanied hydrocephalus (EI > 0.3). Parkinsonism subtypes (PD, MSA, or PSP), AP diameter of the midbrain, and MB^Tegm^ were significantly different among patients with and without hydrocephalus. After regression analysis, parkinsonism subtype stood out to be the most key risk factor of hydrocephalus. The comparison between patients with PSP with and without hydrocephalus did not disclose specific clinical characteristics or risk factors.

**Conclusions:**

This study demonstrates that the presence of NPH‐like MRI features is much higher in PSP patients, and this tendency is decided upon the determination of parkinsonism subtype. Sharing pathophysiological characteristics in these two diseases is implied. More diagnostic tools are needed to better differentiate the two diseases and decide the treatment. To closely observe hydrocephalic parkinsonism patients and well inform the possible limited shunting benefits if PSP core features appear, will be more pivotal and practical at present clinical practice.

## Graphical abstract

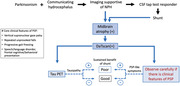

## 1 INTRODUCTION

Idiopathic normal pressure hydrocephalus (NPH) was first described by Hakim and Adams in 1965, characterized by the typical Hakim triad: gait disturbance, cognitive decline, urinary incontinence, and the radiological hallmark of ventriculomegaly ( Adams et al., 1965; Hakim & Adams, 1965). Its prevalence is reported to be 10.2–22 per 100,000 persons and is higher in the elderly, especially those older than age 60 (Martín‐Láez et al., 2016; Kuriyama et al., 2017). According to the criteria adpated from the third edition of Japanese NPH guideline (Nakajima et al., 2021), one symptom in gait/urination/cognition accompanied by ventriculomegaly fits the classification of possible NPH, and gait disturbance is a quite common early presentation of atypical parkinsonism.

On diagnosing NPH, Evans’ index (EI) > 0.3 is the most common and available screening test for ventriculomegaly. Disproportionately enlarged subarachnoid space hydrocephalus (DESH) has been advocated as a diagnostic imaging feature of NPH in the Japanese guideline (Mori et al., 2012; Nakajima et al., 2021); however, its low negative predictive value lessens its clinical application as a diagnostic or prognostic marker (Craven et al., 2016; Nakajima et al., 2021). Callosal angle (CA) is the index that indirectly expresses DESH, and the addition of CA as a supportive radiological parameter helps in differentiating NPH patients from Alzheimer disease (AD) and normally aged subjects (Miskin et al., 2017; Nakajima et al., 2021; Park et al., 2021). The limitation of CA measurement is the great variation in different measurement position and method, and standardizing the measurement protocol is essential (Nakajima et al., 2021). Until now, the shortage of biological markers causes diagnostic dilemma, and NPH has been chronically overlooked by clinicians.

NPH mainly affects the elderly, a population particularly vulnerable to comorbid conditions and neurodegenerative diseases. Based on several prior studies and reviews, NPH is commonly associated with AD, cardiovascular disease, stroke, and vascular risk factors such as diabetes mellitus and hypertension (Israelsson et al., 2017; Williams & Malm, 2016). Besides, Parkinson's disease (PD), dementia with Lewy bodies, corticobasal degeneration (CBD), progressive supranuclear palsy (PSP), and multiple system atrophy (MSA) are important differential diagnosis of NPH (Williams & Malm, 2016). Among them, PSP is the most clinically and radiologically NPH‐mimic disease (Constantinides, et al., 2020; Ohara et al., 2020; Onder et al., 2022; Quattrone et al., 2020). In a study on 50 NPH patients, Pozzi et al. found a symmetric reduction of striatal dopamine reuptake transporter and motor impairments during a 2‐year follow‐up (Pozzi et al., 2021). Some reports also mentioned about the recovery of reduced striatal dopamine transporter density after shunting (Sarica, et al., 2021; Todisco et al., et al. 2021). These reports obscure the delineation between NPH and atypical neurodegenerative Parkinsonian syndromes both phenotypically and pathophysiologically.

Here, the rate of hydrocephalus and other supportive imaging features of NPH were assessed in patients with the diagnosis of PD, MSA, and PSP. The demographic variables among three groups of parkinsonism patients were compared. The aim of this study is to remind clinicians on the imaging characteristic of PSP, as well as the importance of atypical parkinsonism as the possible underlying pathophysiology of NPH.

## 2 MATERIALS AND METHODS

### 2.1 Patients

We retrospectively analyzed and reviewed the clinically diagnosed patients with PD, MSA, and PSP documented in the Center for the Parkinson's disease at Kaohsiung Chang Gung Memorial Hospital between October 2019 and September 2020. Demographic information and clinical information were obtained from medical records, and age at onset was used in the analysis. In all cases, only anonymized information was used for the study. Twenty‐four patients with clinical diagnosis of probable MSA, 34 with probable PSP patients, and 58 sex‐ and age‐matched patients with PD were enrolled. Among the 34 PSP patients, 18 were PSP‐Richardson's syndrome (PSP‐RS) (52.9%), nine were PSP‐parkinsonism (PSP‐P) (26.4%), four were PSP‐corticobasal syndrome (PSP‐CBS) (11.8%), two were PSP‐frontal (PSP‐F) (5.9%), and one was PSP‐progressive gait freezing (PSP‐PGF) (3.0%). Among the 24 patients with MSA, 16 were MSA‐parkinsonism (MSA‐P) (66.7%) and eight were MSA‐cerebellar ataxia (MSA‐C) (33.3%). The diagnosis of PSP and MSA was based on 2017 and 2008 MDS criteria, respectively (Gilman et al., 2008; Höglinger et al., 2017). The score of unified Parkinson's disease rating scale (UPDRS) and mini‐mental state examination (MMSE) was collected. This study was approved by the local Institutional Ethics Committee (IRB No.: 202001765B0).

### 2.2 Magnetic resonance imaging

The magnetic resonance imaging (MRI) data of the 116 patients with PD, MSA, and PSP analyzed in our study were obtained. The first MRI study of each patient was selected for imaging evaluation. The EI was determined on axial view by measuring the largest width of the frontal horns divided by the largest width of the inner skull at the same level. Increased EI > 0.3 is defined as hydrocephalus. In those patients with EI > 0.3, CA was measured on a coronal plane perpendicular to the anterior commissure–posterior commissure (AC‐PC) line at the level of posterior commissure (PC) and between the medial walls of the lateral ventricles. Steep CA is defined as angle < 90° (Nakajima et al., 2021).

The presence of periventricular hyperintensities (PVH) was defined as abnormal white matter hyperintense regions just adjacent to the lateral ventricles through T2‐weighted or fluid‐attenuated inversion recovery (FLAIR) images (Tullberg et al., 2001). Disproportionately enlarged subarachnoid space hydrocephalus (DESH) was defined as “tight high‐convexity subarachnoid spaces along with Sylvian dilatation” and “EI > 0.3” on the imaging (Hashimoto et al., 2010).

Antero‐posterior (AP) diameter of the midbrain at the level of the superior colliculus was measured from the interpeduncular fossa to the anterior margin of the cerebral aqueduct on axial T1‐weighted images or FLAIR images (Righini et al., 2004). The length of the midbrain tegmentum diameter (MB^Tegm^) was measured on an axial T1‐weighted image as previously described. Briefly, an axial image at the level of mid‐mammillary body was chosen, and the distance from the interpeduncular fossa to the center of the aqueduct was measured (Kim et al., 2015). The measurement methods are shown in Figure S1. All analyses were performed by the same rater (Mu‐Hui Fu), who was blinded to the clinical diagnosis and has more than 10 years of neuroimaging training.

### 2.3 Dopamine transporter single photon emission computed tomogram scan (DaTscan)

Ninety‐three of the patients were injected intravenously with a single bolus dose of 740 MBq (20 mCi) 99mTc‐TRODAT‐1. The brain SPECT/CT (Symbia T; Siemens, Erlangen, Germany) images were obtained 4 h later. Regions of interests were drawn on the caudate and putamen of each hemisphere. The occipital cortex served as a background area. The ratio of the specific to nonspecific striatal (caudate and putamen) TRODAT‐1 binding in each region was calculated by mean region of interest (ROI) counts divided by mean occipital cortex counts (Table S1).

### 2.4 Statistical analysis

The difference of categorical variables was analyzed using the chi‐square test, and differences of continuous variables among three groups were analyzed using a one‐way analysis of variance. Scheffe test was used for post‐hoc analysis. In the analysis about different variables between groups of hydrocephalus (EI > 0.3) and non‐hydrocephalus (EI < 0.3), independent‐samples *T* test was used. Finally, logistic regression analysis was used to identify the influence of independent variables on hydrocephalus. In patients with PSP with and without hydrocephalus, chi‐square and nonparametric tests were applied. Statistical significance was set at 𝑝 < .05. All statistical analyses were conducted by using the IBM SPSS software package, version 22 (IBM, Inc., Armonk, NY). All graphs were designed and modified in SPSS software.

## 3 RESULTS

**FIGURE 1:**
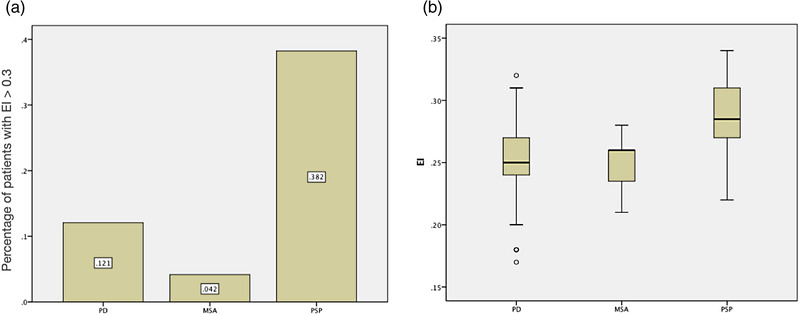
Higher Evans’ index (EI) in patients with progressive supranuclear palsy (PSP). (a) The histogram of ventriculomegaly (EI > 0.3) in three types of parkinsonism. The proportion is shown in the bar. (b) The EI score is significantly higher in patients with PSP

Twenty‐four patients with clinical diagnosis of probable MSA, 34 with probable PSP, and 58 with PD were enrolled. Abnormal presynaptic dopaminergic lesion, symmetric or asymmetric, was proved in TRODAT scan among 93 patients (Table S1). The prevalence rate of hydrocephalus (EI > 0.3) was significantly higher among patients with PSP than the other two groups (PSP: 38.2%, PD: 12.1%, MSA: 4.2%, *p* = .002, Table 1, Figure 1a; Table S1). The mean value of EI among three groups was also significantly higher in patients with PSP (0.29 ± 0.03, *p* < .001, Table 1, Figure 1b).

**TABLE 1:** Demographics, clinical, and MRI data of the three groups of parkinsonism patients

	PD	MSA	PSP	p‐Value	Post hoc significance
Number of subjects	58	24	34		
Sex (M/F)	38/20	12/12	26/8	.064	
EI > 0.3 (No. [%])	7 (12.1%)	1 (4.2%)	13 (38.2%)	.002**	
EI	0.25 (0.03)	0.25 (0.02)	0.29 (0.03)	.000**	PSP>PD, MSA
Age, years	63.95 (10.45)	57.17 (8.27)	71.74 (8.43)	.000**	PSP>PD > MSA
Disease duration, years	2.28 (2.77)	1.42 (1.47)	1.59 (1.65)	.192	
MMSE	24.41 (6.565)	25.20 (5.181)	21.88 (8.282)	.320	
UPDRS (part III)	25.40 (7.882)	28.13 (13.281)	36.92 (12.861)	.000**	PSP>PD, MSA
AP diameter of midbrain	15.53 (1.4)	15.81 (1.3)	13.99 (1.3)	.000**	PSP<PD, MSA
MB^Tegm^	11.97 (1.07)	11.81 (0.99)	10.22 (0.98)	.000**	PSP<PD, MSA
DESH (No. [%])	0	0	6 (17.6%)	.000**	
PVH (No. [%])	8 (13.8%)	3 (12.5%)	21 (61.8%)	.000**	

*Note*: All data are presented as mean (SD) except number of subjects, EI > 0.3, sex, DESH, and PVH.

Abbreviations: AP, antero‐posterior; DESH, disproportionately enlarged subarachnoid space hydrocephalus; EI, Evans’ index; F, female; MB^Tegm^, midbrain tegmentum diameter; M, male; MSA, multiple system atrophy; MMSE, mini‐mental state examination; PD, Parkinson's disease; PSP, progressive supranuclear palsy; PVH, periventricular hyperintensities; UPDRS, unified Parkinson's disease rating scale; SD: standard deviation. *p < .05; **p < .01.

The demographic data on three groups of patients are listed in the Table 1. The age in patients with PSP was statistically older than those with PD or with MSA (PSP: 71.74 ± 8.43, PD: 63.95 ± 10.45, MSA: 57.17 ± 8.27, *p* < .001). The UPDRS part III motor score was significantly higher in patients with PSP. There were no significant differences among groups regarding sex and disease duration. The rate of hydrocephalus, PVH, and DESH was higher in patients with PSP than in other two groups. MRI features of PSP, such as AP diameter of midbrain and MB^Tegm^, have shown to be smaller in patients with PSP (*p* < .001).

Demographic data on hydrocephalus (EI > 0.3) and non‐hydrocephalus (EI < 0.3) patients among the three groups are shown in Table 2. The proportion of hydrocephalus was 38.2% in PSP, 12.1% in PD, and 4.2% in MSA (*p* = .002). There were no significant differences in the sex and disease duration between patients with and without hydrocephalus. Patients with hydrocephalus were significantly older than those without. The MRI features, including AP diameter of midbrain and MB^Tegm^, were significantly shorter in patients with hydrocephalus (Table 2, *p* = .015, *p* = .003, respectively). The mean CA of PSP with EI > 0.3 was 115.785 ± 17.9996°, and that of PD was 108.271 ± 16.568°. Steep CA was found in two of PSP (No. 2 and 22) and one of PD (No. 34) hydrocephalic patients. The CA of the only one EI > 0.3 MSA patient was 120° (Table S1).

**TABLE 2:** Comparison of demographic, clinical, and MRI data between groups of hydrocephalus and non‐hydrocephalus

		EI		p‐Value
		>0.3	<0.3	
Total numbers		21	95	
Subtypes	PD	7	51	.002**
	MSA	1	23	
	PSP	13	21	
Sex (M/F)		17/4	59/36	.054
Age, years		72.05 (7.59)	63.23 (10.68)	.001**
Disease duration, years		1.62 (1.88)	1.96 (2.36)	.539
AP diameter of midbrain		14.41 (1.56)	15.29 (1.47)	.015*
MB^Tegm^		10.68 (1.25)	11.59 (1.24)	.003**

*Note*: All data are presented as mean (SD).

Abbreviations: AP, antero‐posterior; EI, Evans’ index; MB^Tegm^, midbrain tegmentum diameter; MSA, multiple system atrophy; PD, Parkinson's disease; PSP, progressive supranuclear palsy.

*p < .05; **p < .01.

The analysis on patients with PSP with and without hydrocephalus is depicted in Table S2. The parameters include sex, onset age, disease duration, and clinical symptoms, and none was found to be significantly different between the two groups of patients.

We further stratified the different risk factors of hydrocephalus with logistic regression analysis. Forward conditional method was applied according to the results of univariate analysis. The significant univariate factors and possible confounding factors used in logistic regression included subtype, AP diameter of midbrain, and MB^Tegm^. Age was not included in the analysis due to high degree of multicollinearity with the classification of parkinsonism subtypes. After analyzing all the above‐mentioned variables, only subtype was independently associated with the presence of hydrocephalus (Table 3).

**TABLE 3:** Logistic regression analysis of risk factors for hydrocephalus

Significant univariate factors	Adjusted OR of hydrocephalus (95% CI)	p‐Value
Subtypes		.003**
Subtypes (PD‐PSP)	0.22 (0.078–0.634)	.005**
Subtypes (MSA‐PSP)	0.07 (0.008–0.584)	.014*
AP diameter of midbrain		.455
MB^Tegm^		.295

*Note*: Subtypes and age are two variables with multicollinearity.

Abbreviations: AP, antero‐posterior; CI: confidence interval; MB^Tegm^, midbrain tegmentum diameter; OR: odds ratio.

*p < .05; **p < .01.

## 4 DISCUSSION

The high co‐occurrence rate between NPH and neurodegenerative disorders has been reported (Williams & Malm, 2016; Espay et al., 2017), among them atypical parkinsonism bears most resemblance to NPH regarding their overlapped clinical spectrum. PSP shares common features with NPH, particularly in patients lacking vertical gaze palsy. Though increasing evidence demonstrates the similarities between these two diseases, no studies till now has focused on the hydrocephalic presentation from the perspective of different Parkinsonian syndromes. In our study, the incidence of hydrocephalus was significantly higher in PSP (38.2%) patients than that in patients with PD (12.1%) or MSA (4.2%). The results also suggest that the rate of hydrocephalus is determined, while the diagnosis of parkinsonism subtype is made; thus, the demarcation between NPH and PSP seems blurring and difficult to distinguish one from another.

Our findings support the results of prior studies about the co‐occurrence of NPH in PSP cases. From the experience of Queen Square Brain Bank and University of Cincinnati, three out of four presumed NPH cases were proved to be PSP after autopsy (Magdalinou et al., 2013; Starr et al., 2014). Thirty‐three out of 87 patients with PSP (37.9%) had ventricular dilatation in Sarica et al.’s (2021) study. The imaging feature of PSP‐hummingbird sign, has ever been reported as a supportive diagnosis of NPH. (Atalay et al., 2021). The report from Ohara and colleagues even proposed a clinical phenotype of PSP with hydrocephalus (Ohara et al., 2020). Traditionally, NPH and PSP are classified as two distinct diseases and should be thoroughly distinguished to avoid futile treatment. Several researchers tried to find additional MRI indexes differentiating one from the other, such as Magnetic Resonance Parkinsonism Index, Magnetic Resonance Hydrocephalic Index, interpeduncular angle, midbrain area, and cortical thickness (Bianco et al., 2022; Ohara et al., 2020; Quattrone et al., 2020; Ugga et al., 2020; Virhammar et al., 2022). In our opinion, a portion of the two entities does overlap and share similar features, implying the transitory role of NPH mimics in the clinical course of PSP.

In our study, steep CA was found in two of PSP and one of PD hydrocephalic patients. In fact, after reviewing the chart of No. 34 PD patient in the later follow‐up, she fits the diagnostic criteria of PSP. This finding again supports the hydrocephalus as the unique imaging feature of PSP. Though differentiating NPH from PSP may provide more precise prediction on therapeutic prognosis, several reports have demonstrated that shunting can achieve transient response in PSP with NPH‐like radiologic features. (Morariu, 1979; Magdalinou et al., 2013; Onder, 2020). Most of the additional imaging indexes require specific settings or analysis; thus, wide clinical application is limited. Though yielding low specificity, the convenience of EI measurement as the first step of hydrocephalus screening is undeniable. Addition of other imaging markers can aid the diagnosis of NPH, but these should not be regarded as the prerequisite for shunt surgery, an opinion that we agree with a group of Sweden radiologists (Agerskov et al., 2019).

PSP is a primary four‐repeat tauopathy disease characterized by tau protein accumulation in neurons and glia, forming “neurofibrillary tangles” and “tufted astrocytes” (Shoeibi et al., 2019). Tau is a microtubule‐associated protein expressing in neurons, and functions as the axonal microtubule stabilizer. However, while this protein becomes pathologic, such as aggregated and/or hyperphosphorylated, it accumulates inside other cells such as astrocytes and microglia (Ferrer et al., 2018). Glial cells do not produce Tau, but reports on astrocytes uptaking and internalizing the pathological tau are mounting (Perea et al., 2019). Astrocytes play key roles in the glymphatic system as aquaporin‐4 (AQP4) water channels lining along astroglial endfeet, and this system was proved capable of clearing extracellular tau from the central nervous system (Patel et al., 2019). The contribution of tau in the glymphatic system and the formation of hydrocephalus worth further evaluation.

There are some limitations in this study. First, the high multicollinearity exists between age and parkinsonism subtypes. It is well known that the incidence of NPH increases with age. The onset of MSA usually developed in younger age at around 50−60 years (Fanciulli & Wenning, 2015), while the onset of PSP is 60–70 years (McFarland, 2016). The multiple underlying diseases in the elderly may pose these patients under the threat of disturbed CSF flow, and more vulnerable to the formation of hydrocephalus. However, since the exact cause of hydrocephalus in NPH remains uncertain, the contribution of each aging‐related factor to hydrocephalus is difficult to determine. Moreover, it is evident that the proportion of hydrocephalus in PSP greatly outnumbered that in MSA and PD, a finding being too conspicuous to ignore the role of parkinsonism subtype in hydrocephalus. Second, the design of our study is retrospective cross‐sectional. Longitudinal prospective study is required to identify the exact proportion of hydrocephalic manifestations in patients with PSP and provides us more accurate data. Third, all radiological analyses were performed by the same rater, and the reliability of interpretation would increase if inspected by more raters. Lastly, the definite diagnosis of atypical parkinsonism classification cannot be achieved due to lacking histopathologic evidence.

Our study shows that NPH‐like radiological features are more common in PSP than in other neurodegenerative parkinsonism. Though differentiating one from another might be helpful for therapeutic decision and prognosis prediction, the diagnosis of PSP should not preclude patient from the choice of shunting for still transient improvement being reported. However, to predict a more sustainable benefit of shunting, more tools are certainly required. With the advent and improvement of tau‐specific ligands, tau positron emission tomography (PET) is emerging as a potential biomarker in the diagnosis of PSP (Schröter et al., 2020; Tagai et al., 2021).

**FIGURE 2:**
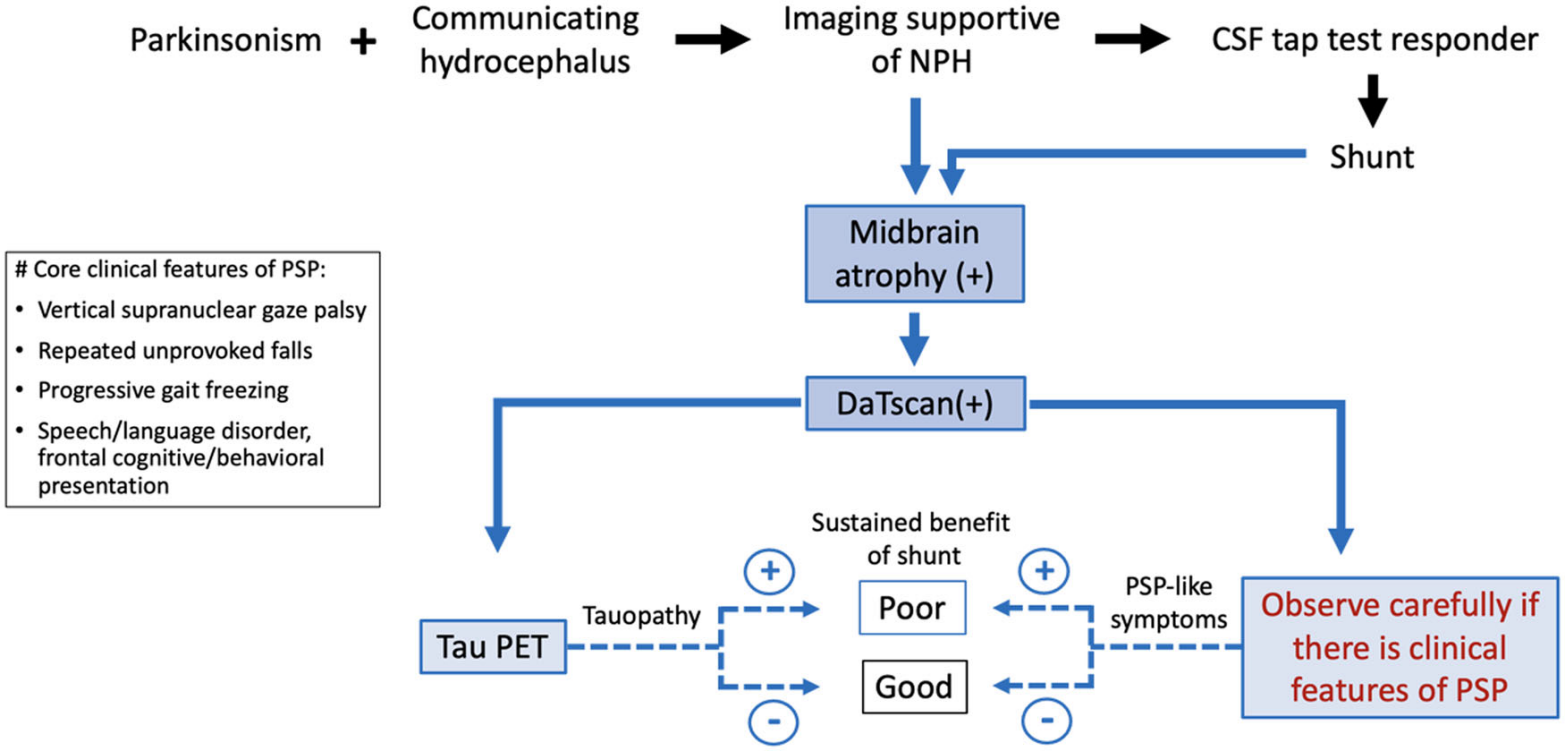
Proposed diagnostic and management flow chart for patients with parkinsonism and normal pressure hydrocephalus (NPH)‐like imaging features. The reference of progressive supranuclear palsy (PSP) core features is from 2017 International Parkinson and Movement Disorder Society (Höglinger et al., 2017). Black arrows are the traditional management process. Blue arrows are our proposal for NPH‐like patients: solid line means strong recommendation, and dashed line means suggestion. This predictive paradigm of long‐term shunt response is also applicable for patients having received shunt placement. “Midbrain atrophy” is defined as midbrain antero‐posterior (AP) diameter <13.5 mm (Warmuth‐Metz et al., 2021). DaTscan(+) means decreased dopamine transporter density in bilateral basal ganglia (Wallert et al., 2022). Abbreviations: DaTscan, dopamine transporter single photon emission computed tomogram scan; PET, positron emission tomography

Figure 2 depicts our suggestion on scouting PSP‐like features while approaching or following a parkinsonism patient with hydrocephalus. In this clinically oriented flowchart, we propose “midbrain atrophy” as a *neuroimaging* marker in NPH patients, “closely observe if there is PSP‐like feature” from the *clinical* perspective, and “Tau PET” (if accessible) from the *biochemical* direction. We anticipate predicting a more sustainable shunting benefit through these three clinical approaching steps, and this paradigm is also applicable for patients having received shunt placement.

## 5 CONCLUSION

Our study shows that NPH‐like radiologic features are more prevalent in PSP patients. A portion of NPH and PSP patients present high similarities not only in clinical manifestations but also in imaging appearances upon diagnosis. Differentiating one from another might be helpful to decide the therapeutic method, but we certainly need more biological imaging tools other than MRI to assist the diagnosis in the future. Closely observe patients with hydrocephalic parkinsonism and well inform the possible limited shunting benefits if PSP core features develop will be more pivotal and practical at present.

## CONFLICT OF INTEREST

The authors declare no conflict of interest.

### PEER REVIEW

The peer review history for this article is available at https://publons.com/publon/10.1002/brb3.2884

## Supporting information

Supp Information

## Data Availability

The authors confirm that the data support the findings of this study are available within the article and its supporting information.
