# Supplementary material for: Higher prevalence of idiopathic normal pressure hydrocephalus‐like MRI features in progressive supranuclear palsy: An imaging reminder of atypical parkinsonism
Source: Brain Behav. 2023 Jan 12;13(2):e2884. doi: 10.1002/brb3.2884 (PMC9927835; doi:10.1002/brb3.2884)
Supplement: Supplementary file 1 — Supp Information [file BRB3-13-e2884-s001.docx]

**Supplementary Figure 1. Imaging parameter measurements.**

**
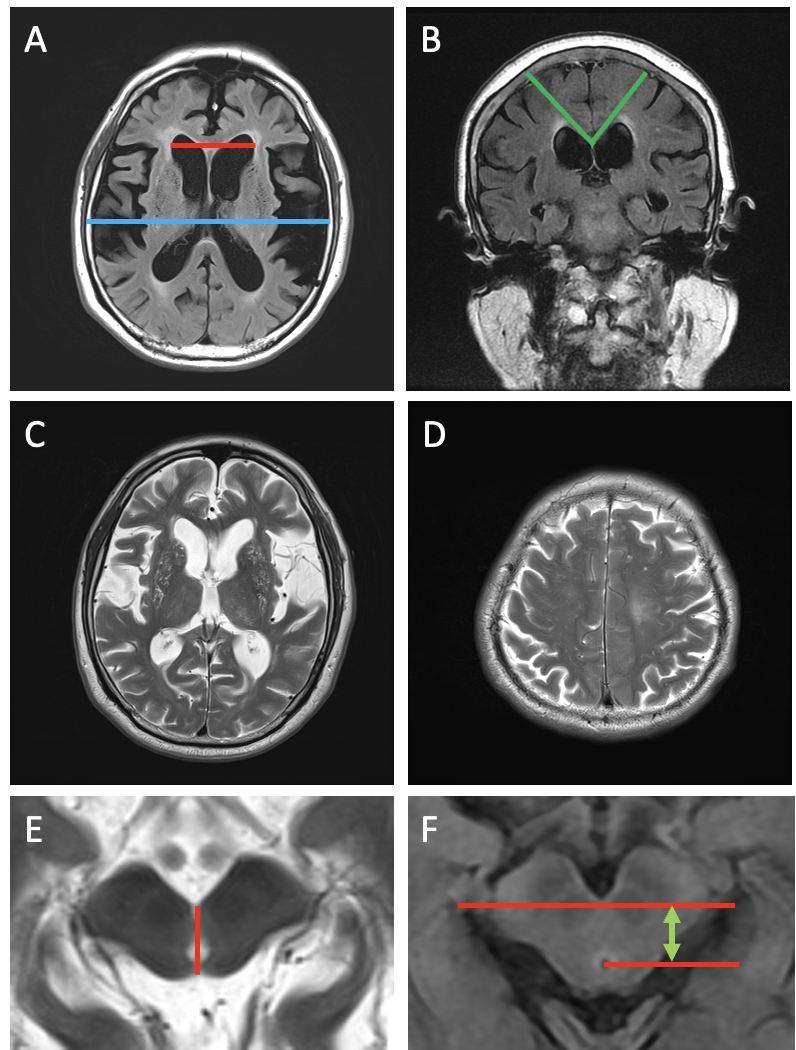
**

1. Evans’ index (EI)= red line/blue line.
2. Callosal angle: the angle between two green lines.

(C, D) Disproportionately enlarged subarachnoid space hydrocephalus (DESH): enlargement of CSF spaces in the Sylvian fissures (C) but crowded in the sulci superiorly near the vertex (D).

(E) Antero-posterior diameter of the midbrain: red line length. (Righini, Antonini et al. 2004)

(F) Midbrain tegmentum diameter (MB^Tegm^): Green double-headed arrows length. (Kim, Ma et al. 2015)

**Supplementary Table 1. The raw imaging parameters in three groups of parkinsonism patients**.

**# MSA**

| **No.** | **Sex** | **Age** | **Evans’ index**  (Diameter of the frontal horns/Diameter of widest brain)  (mm) | **Callosal angle (age)** | **TRODAT**  (Striatal/occipital cortex ratio, R’t/L’t) |
| --- | --- | --- | --- | --- | --- |
| 1 | M | 45 | 0.26 |  | 1.25/1.41 |
| 2 | M | 67 | 0.26 |  | NA |
| 3 | F | 64 | 0.23 |  | 1.79/1.99 |
| 4 | M | 57 | 0.25 |  | 1.43/1.57 |
| 5 | M | 73 | 0.26 |  | 1.60/1.65 |
| 6 | F | 68 | 0.26 |  | NA |
| 7 | F | 60 | 0.25 |  | 1.53/1.57 |
| 8 | F | 64 | 0.24 |  | 1.78/1.72 |
| 9 | M | 51 | 0.22 |  | 1.58/1.31 |
| 10 | M | 62 | 0.23 |  | NA |
| 11 | F | 50 | 0.21 |  | 1.57/1.96 |
| 12 | F | 59 | 0.27 |  | NA |
| 13 | F | 51 | 0.30 | 120.1º (59) | NA |
| 14 | M | 54 | 0.24 |  | 1.7/1.8 |
| 15 | F | 48 | 0.23 |  | 1.43/1.42 |
| 16 | M | 57 | 0.26 |  | 1.40/1.34 |
| 17 | M | 42 | 0.24 |  | 1.64/1.63 |
| 18 | F | 44 | 0.22 |  | NA |
| 19 | F | 61 | 0.27 |  | 1.74/1.67 |
| 20 | M | 46 | 0.27 |  | 1.46/1.48 |
| 21 | M | 60 | 0.26 |  | 1.36/1.41 |
| 22 | F | 65 | 0.27 |  | NA |
| 23 | F | 55 | 0.26 |  | NA |
| 24 | M | 64 | 0.26 |  | 1.5/1.5 |

**#PSP**

| **No.** | **Sex** | **Age** | **Evans’ index**  (Diameter of the frontal horns/Diameter of widest brain)  (mm) | **Callosal angle (age)** | **TRODAT**  (Striatal/occipital cortex ratio, R’t/L’t) |
| --- | --- | --- | --- | --- | --- |
| 1 | M | 68 | 0.27 |  | 1.62/1.61 |
| 2 | M | 68 | 0.33 | 78.1º (81) | 1.08/1.16 |
| 3 | M | 78 | 0.34 | 124º (73) | 1.11/1.29 |
| 4 | F | 63 | 0.26 |  | 1.25/1.21 |
| 5 | F | 73 | 0.31 | 124º (79) | 1.2/1.13 |
| 6 | F | 57 | 0.25 |  | NA |
| 7 | F | 67 | 0.28 |  | 1.2/1.2 |
| 8 | M | 68 | 0.31 | 114º (73) | 1.18/1.1 |
| 9 | M | 83 | 0.29 |  | 1.4/1.4 |
| 10 | M | 70 | 0.27 |  | 1.06/1.11 |
| 11 | M | 71 | 0.32 | 126.8º (73) | 1.71/1.74 |
| 12 | M | 76 | 0.33 | 114.9º (76) | 1.27/1.29 |
| 13 | M | 78 | 0.34 | 96.5º (80) | 1.49/1.12 |
| 14 | M | 78 | 0.32 | 126.9º | 1.5/1.5 |
| 15 | M | 62 | 0.25 |  | NA |
| 16 | M | 69 | 0.31 | 127.8º (70) | 1.23/1.36 |
| 17 | M | 57 | 0.28 |  | 1.14/1.23 |
| 18 | M | 67 | 0.27 |  | 1.5/1.6 |
| 19 | M | 71 | 0.24 |  | NA |
| 20 | F | 82 | 0.28 |  | 1.24/1.25 |
| 21 | M | 77 | 0.28 |  | 1.27/1.55 |
| 22 | M | 57 | 0.31 | 82.9º (64) | 1.29/1.45 |
| 23 | M | 74 | 0.29 |  | 1.37/1.44 |
| 24 | F | 74 | 0.28 |  | 1.23/1.23 |
| 25 | M | 76 | 0.32 | 124.1º (75) | 1.7/1.7 |
| 26 | M | 71 | 0.29 |  | NA |
| 27 | M | 88 | 0.29 |  | 1.29/1.31 |
| 28 | M | 61 | 0.29 |  | NA |
| 29 | F | 83 | 0.28 |  | NA |
| 30 | M | 73 | 0.29 |  | 1.42/1.37 |
| 31 | M | 61 | 0.28 |  | 1.19/1.25 |
| 32 | F | 85 | 0.28 |  | 1.5/1.5 |
| 33 | M | 71 | 0.32 | 130º (72) | 1.12/1.12 |
| 34 | M | 75 | 0.30 | 130.2º (75) | 1.37/1.34 |

**# PD**

| **No.** | **Sex** | **Age** | **Evan’s index**  (Diameter of the frontal horns/Diameter of widest brain)  (mm) | **Callosal angle (age)** | **TRODAT**  (Striatal/occipital cortex ratio, R’t/L’t) |
| --- | --- | --- | --- | --- | --- |
| 1 | M | 72 | 0.26 |  | 1.33/1.25 |
| 2 | M | 45 | 0.21 |  | 1.35/1.32 |
| 3 | M | 71 | 0.25 |  | 1.5/1.5 |
| 4 | M | 54 | 0.25 |  | 1.43/1.44 |
| 5 | M | 68 | 0.17 |  | 1.26/1.25 |
| 6 | F | 65 | 0.24 |  | 1.5/1.5 |
| 7 | F | 60 | 0.26 |  | NA |
| 8 | F | 72 | 0.23 |  | 1.65/1.63 |
| 9 | F | 62 | 0.27 |  | 1.31/1.47 |
| 10 | M | 74 | 0.26 |  | 1.6/1.7 |
| 11 | M | 67 | 0.25 |  | 1.52/1.55 |
| 12 | M | 60 | 0.24 |  | 1.43/1.42 |
| 13 | M | 52 | 0.27 |  | NA |
| 14 | M | 68 | 0.26 |  | NA |
| 15 | M | 74 | 0.20 |  | 1.32/1.44 |
| 16 | F | 60 | 0.26 |  | 1.44/1.51 |
| 17 | M | 57 | 0.24 |  | 1.37/1.32 |
| 18 | M | 41 | 0.24 |  | 1.47/1.25 |
| 19 | F | 64 | 0.24 |  | 1.75/1.66 |
| 20 | M | 69 | 0.24 |  | 1.27/1.20 |
| 21 | F | 70 | 0.26 |  | 1.5/1.4 |
| 22 | M | 68 | 0.25 |  | 1.25/1.29 |
| 23 | F | 59 | 0.23 |  | 1.34/1.25 |
| 24 | M | 75 | 0.26 |  | 1.41/1.22 |
| 25 | M | 59 | 0.18 |  | 1.36/1.34 |
| 26 | F | 46 | 0.25 |  | 1.44/1.47 |
| 27 | F | 64 | 0.18 |  | 1.8/1.6 |
| 28 | M | 80 | 0.30 | 131.9º (80) | NA |
| 29 | M | 81 | 0.28 |  | 1.35/1.37 |
| 30 | F | 50 | 0.23 |  | NA |
| 31 | M | 62 | 0.26 |  | 1.25/1.28 |
| 32 | M | 66 | 0.28 |  | 1.33/1.23 |
| 33 | F | 61 | 0.24 |  | 1.43/1.44 |
| 34 | F | 71 | 0.32 | 88.6º (81) | 1.46/1.49 |
| 35 | F | 67 | 0.27 |  | 1.22/1.29 |
| 36 | M | 64 | 0.29 |  | 1.5/1.42 |
| 37 | M | 46 | 0.25 |  | 1.53/1.64 |
| 38 | M | 77 | 0.30 | 124.8º (77) | 1.27/1.16 |
| 39 | M | 73 | 0.25 |  | 1.5/1.5 |
| 40 | M | 67 | 0.26 |  | 1.3/1.24 |
| 41 | F | 80 | 0.25 |  | 1.29/1.22 |
| 42 | M | 73 | 0.32 | 92.8º (81) | 1.5/1.6 |
| 43 | M | 65 | 0.29 |  | 1.02/1.11 |
| 44 | M | 80 | 0.27 |  | NA |
| 45 | M | 44 | 0.24 |  | 1.64/1.57 |
| 46 | M | 63 | 0.22 |  | 1.07/1.18 |
| 47 | M | 76 | 0.29 |  | 1.4/1.5 |
| 48 | M | 74 | 0.32 | 93.9º (73) | NA |
| 49 | M | 73 | 0.25 |  | 1.7/1.5 |
| 50 | F | 83 | 0.32 | 114º (83) | 1.23/1.22 |
| 51 | M | 61 | 0.31 | 121.5º (61) | NA |
| 52 | F | 53 | 0.27 |  | 1.96/2 |
| 53 | F | 45 | 0.24 |  | 2.04/2.13 |
| 54 | F | 55 | 0.25 |  | 1.33/1.22 |
| 55 | M | 55 | 0.26 |  | 1.4/1.3 |
| 56 | M | 41 | 0.18 |  | 1.31/1.30 |
| 57 | M | 55 | 0.25 |  | 1.27/1.31 |
| 58 | F | 67 | 0.27 |  | 1.29/1.24 |

M: male; F: female; NA: not available

EI **≥** 0.3 is marked as red, and CA < 90º is marked as green.

**Supplementary Table 2. Demographics of PSP patients with and without hydrocephalus.**

|  | EI | | *P*-value | |
| --- | --- | --- | --- | --- |
|  | > 0.3 | < 0.3 | |  |
| Total numbers | 13 | 21 | |  |
| Sex (M/F) | 12/1 | 14/7 | | 0.116 |
| Onset age, years | 71.38 (5.752) | 69.24 (9.643) | | 0.198 |
| Disease duration, months | 33.46 (34.719) | 36.95 (25.897) | | 0.297 |
| Vertical gaze palsy (+/-) | 6/7 | 12/9 | | 0.455 |
| Postural instability (+/-) | 8/5 | 15/6 | | 0.709 |
| Cognitive impairment (+/-) | 8/5 | 9/12 | | 0.481 |

All data are presented as mean (SD). **P* < 0.05.
